# Radium-223 for the Management of Bone Metastases in Castration-Resistant Prostate Cancer

**Published:** 2015-11-01

**Authors:** Heather Cox, Megan Hames, Mona Benrashid

**Affiliations:** Novant Health Oncology Specialists, Greensboro/Winston-Salem, North Carolina; Vanderbilt University Medical Center, Nashville, Tennessee

In the United States, prostate cancer is the most common cancer and second leading cause of cancer-related deaths in men. It is estimated that 220,800 new cases of prostate cancer will have been diagnosed in 2015, with an estimated 27,540 deaths (American Cancer Society, 2015).

Although prostate cancer is typically diagnosed when it is localized and considered curable, approximately 4% of patients have metastatic cancer at diagnosis. For androgen-sensitive metastatic disease, the current standard of care is continuous androgen-deprivation therapy (National Comprehensive Cancer Network, 2015). However, a large proportion of patients progress to metastatic castration-resistant prostate cancer (CRPC) and require additional therapy.

Over 90% percent of patients with metastatic CRPC develop bone metastases (Bubendorf et al., 2000). As the vast majority of patients with metastatic CRPC have bone metastases, there is an increase in morbidity and mortality due to skeletal-related events. Over the past decade, several treatment options have demonstrated a survival benefit in metastatic CRPC, including abiraterone (Zytiga), docetaxel, cabazitaxel (Jevtana), sipuleucel-T (Provenge), and enzalutamide (Xtandi; Wissing, van Leeuwen, van der Pluijm, & Gelderblom, 2013). Radium-223 dichloride (Xofigo) is a newly approved agent that specifically targets bone metastases in metastatic CRPC.

## RADIOPHARMACEUTICALS

Radiopharmaceuticals target bone by binding to hydroxyapatite, the main component of inorganic matrix in bone that is linked with cancer cells found in osteoblastic lesions. Beta-emitting radiopharmaceuticals (strontium-89 [Sr-89] and samarium-153 [Sm-153]) are approved by the US Food and Drug Administration (FDA) for palliative treatment of bone metastases in patients with metastatic CRPC. Fifty percent of patients show symptomatic relief with treatment but with limited duration of response (Sartor et al., 2004; Serafini, 2000). Due to tissue penetration and track lengths, surrounding tissues can be damaged along with the tumor cells. This can result in significant adverse effects, including bone marrow toxicity. Despite significant palliative benefits in patients with bone metastases, the beta-emitting particles have failed to show a survival benefit (Bellmunt, 2013).

Radium-223 is the only currently available alpha-emitting radiopharmaceutical. The penetration of alpha particles is approximately 40–100 m (2–10 tumor cell diameters), whereas Sr-89 and Sm-153 have a tissue penetration of approximately 2.4 and 0.6 mm, respectively Bellmunt, 2013). The short-track length of radium-223 infers limited hematologic toxicity (Lewis & Sartor, 2012).

## MECHANISM OF ACTION

Radium-223 is a calcium mimetic that becomes part of the bone volume over time. Radium-223 forms complexes with hydroxyapatite at areas of increased bone turnover, including bone metastases (Bayer HealthCare Pharmaceuticals, 2013). Radium-223 causes double-strand DNA breaks due to high linear energy transfer via alpha emission, resulting in an antitumor effect on bone metastases.

In metastatic CRPC, the majority of bone metastases are characterized by blastic formation of new bone, although there are components of lytic bone destruction seen as well (Lange & Vessella, 1998). Radium-223 should have a high affinity for osteoblastic bone metastases, as radioactive particles are known to concentrate in areas of increased bone turnover (Humm, Sartor, Parker, Bruland, & Macklis, 2015).

## CLINICAL STUDIES

To date, there have been several studies evaluating the efficacy of radium-223 for the treatment of bone metastases in the setting of metastatic CRPC. The largest of these studies is the ALSYMPCA trial, an international, prospective, double-blind, placebo-controlled phase III study in adult males with metastatic CRPC. Patients were randomized in a 2:1 fashion to receive radium-223 plus best standard of care (BSC) or placebo plus BSC (Parker et al., 2013). Inclusion and exclusion criteria for the ALSYMPCA trial can be found in Table 1.

**Table 1:**
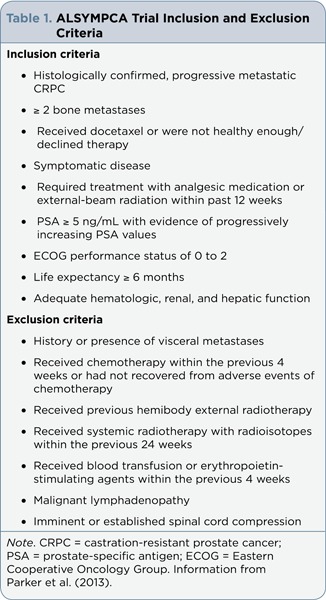
ALSYMPCA Trial Inclusion and Exclusion Criteria

A total of 614 patients received IV radium-223 at a dose of 50 kBq/kg every 4 weeks, and 307 patients were randomized to receive placebo. The study investigators found a statistically significant difference in the primary endpoint, median overall survival (OS). In the updated analysis, patients who received radium-223 plus BSC had a longer median OS, 14.9 months, than patients who received placebo plus BSC, 11.3 months (hazard ratio [HR] = 0.70; 95% confidence interval [CI], 0.58–0.83; p < .001). There was a 30% reduction in the risk of death in patients treated with radium-223 and BSC vs. patients receiving placebo and BSC (Parker et al., 2013).

Additionally, radium-223 plus BSC demonstrated a benefit over placebo plus BSC in all main secondary efficacy endpoints. Patients receiving radium-223 plus BSC had a longer time to the first symptomatic skeletal event, median 15.6 months, than patients who received placebo plus BSC, 9.8 months (HR = 0.66; 95% CI, 0.52–0.83; *p* < .001). Patients receiving radium-223 plus BSC also had a significantly prolonged time to an increase in the total alkaline phosphatase level and time to an increase in the prostate-specific antigen (PSA) level. Lastly, a significantly higher proportion of patients receiving radium-223 plus BSC had a response according to the total alkaline phosphatase level and normalization of this level (Parker et al., 2013).

This study also included a quality-of-life (QOL) assessment utilizing the FACT–P (Functional Assessment of Cancer Therapy–Prostate) total score. A significantly higher percentage of patients who received radium-223 plus BSC had a meaningful improvement in QOL during the period of study drug administration, 25% vs. 16% (*p* = .02) defined as an increase in the FACT–P total score by ≥ 10. There was also a statistically significant mean change in the FACT–P total score in patients receiving radium-223 plus BSC, –2.7 vs. –6.8 (*p* = .006; Parker et al., 2013).

## SAFETY AND TOLERABILITY

The most common adverse drug reactions observed in > 10% of patients who received radium-223 in the ALSYMPCA trial were nausea, diarrhea, vomiting, and peripheral edema. Radiopharmaceuticals targeting bone metastases may damage surrounding tissue, including the bone marrow. Table 2 highlights the hematologic toxicities that were observed in the ALSYMPCA trial. The number of patients experiencing adverse events after receiving the study drug was lower in the radium-223 group than in the placebo group, 93% vs. 96% (Parker et al., 2013; Bayer HealthCare Pharmaceuticals, 2013).

**Table 2:**
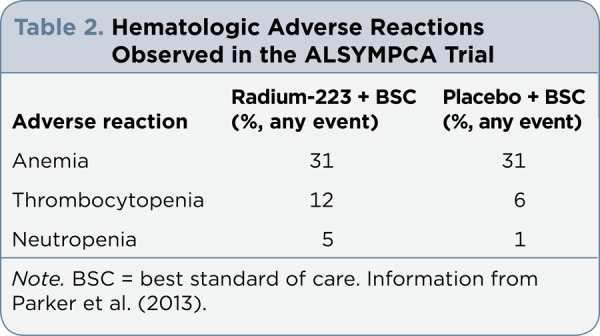
Hematologic Adverse Reactions Observed in the ALSYMPCA Trial

As demonstrated in Table 2, there was no significant difference in the rates of hematologic toxicities when using radium-223 plus BSC and placebo plus BSC. Only one grade 5 hematologic adverse event of thrombocytopenia was considered to be possibly related to the study drug, although the patient did not experience any signs or symptoms of bleeding (Parker et al., 2013).

## DOSING

The FDA-approved dose of radium-223 is 50 kBq (1.35 microcurie) per kilogram of body weight IV every 4 weeks for 6 doses. Radium-223 should be discontinued if dose delays occur due to toxicity, and the dose is held for > 8 weeks (Bayer HealthCare Pharmaceuticals, 2013).

## IMPLICATIONS FOR USE IN CLINICAL PRACTICE

Radium-223 is the first radiopharmaceutical approved by the FDA for the treatment of bone metastases with a significant improvement in median OS. Due to the unique nature of radium-223, the potential for toxicity, and the need for appropriate patient selection, advanced practitioners will need to understand its place in therapy.

Radium-223 has been incorporated into the National Comprehensive Cancer Network (NCCN) and the American Society of Clinical Oncology (ASCO) metastatic CRPC treatment guidelines as an option for patients with symptomatic bone metastases (Basch et al., 2014; NCCN, 2015). In men who have received, are ineligible for, or want to avoid cytotoxic chemotherapy, radium-223 may be a viable treatment option. Radium-223 should not be used in patients with visceral metastases or bulky nodal disease (defined as greater than 3–4 cm), because it has not been shown to extend survival alone in this patient population (Sartor, 2012). Based on data from the ALSYMPCA trial subgroup analyses, prescribers can consider reserving radium-223 for patients who have an increased level of alkaline phosphatase and/or have at least six bone metastases (Parker et al., 2013).

Radium-223 must be administered by an appropriately licensed facility and is usually given either in nuclear medicine or radiation therapy. Important practical considerations for prescribers using radium-223 include ensuring appropriate hematologic evaluation is performed at baseline and prior to each scheduled dose. In addition, providers need to ensure that patients and caregivers receive appropriate counseling on maintaining adequate hydration and radiation safeguards (Bayer HealthCare Pharmaceuticals, 2013). More information on monitoring parameters and patient counseling is provided in Tables 3 and 4.

**Table 3:**
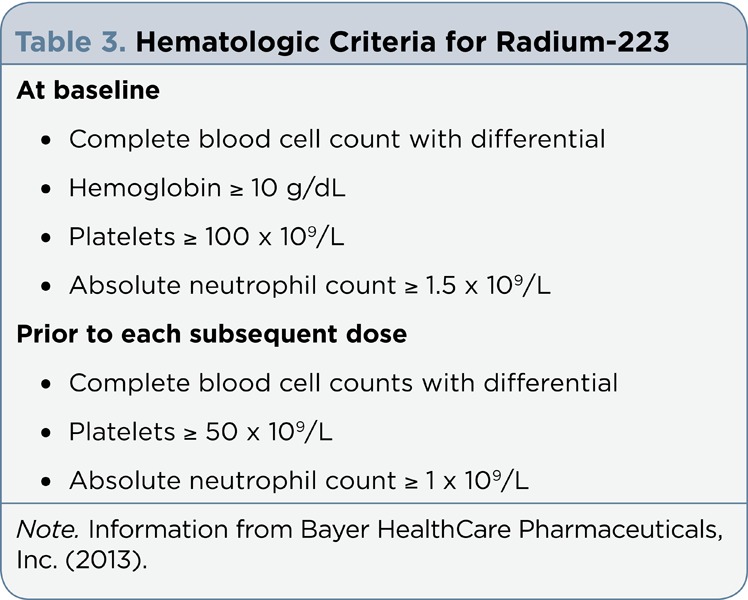
Hematologic Criteria for Radium-223

**Table 4:**
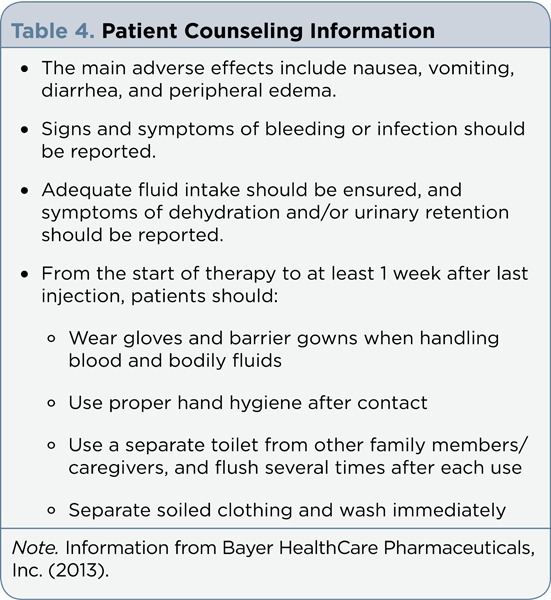
Patient Counseling Information

The cost of radium-223 can be considerable, with the average wholesale price of approximately $12,220 per dose for a 70-kg patient (assuming 0 days from reference date; Lexi-Comp, 2015). Assistance programs are available for those who qualify through Xofigo Access Services (find at XofigoAccesonline.com).

As previously stated, radium-223 is commonly used with androgen-deprivation therapy. However, there are no data to support its use in combination with other agents, and due to the potential for increased myelosuppression, other concomitant therapy should only be administered in the setting of a clinical trial (Bayer HealthCare Pharmaceuticals, 2013). Denosumab (Xgeva) and zoledronic acid do not appear to compete with radium-223 for uptake in the bone and therefore can be used concomitantly (Storto et al., 2006; Lam et al., 2008; James et al., 2013).

Further studies have been requested by the FDA to assess long-term safety as well as optimal dosing. Additional studies may evaluate the efficacy of radium-223 in patients with bone metastases from other tumor types. Studies that combine radium-223 with docetaxel, abiraterone, or enzalutamide are ongoing. Additionally, further studies are needed to evaluate QOL and cost-effectiveness of the available agents and combinations. Therefore, optimal sequencing of medications for metastatic CRPC is unknown, and therapeutic choices should be made based on individual patient characteristics, cost, and patient preference.

## SUMMARY

Treatment options that specifically target bone metastases in patients with metastatic CRPC are needed, as bone metastases are associated with significant morbidity and mortality. Radium-223 represents the first radiopharmaceutical agent with a demonstrated OS benefit. With its manageable toxicity profile, particularly its lack of increased hematologic toxicity vs. placebo, radium-223 provides oncology advanced practitioners with an FDA-approved option for patients with metastatic CRPC and bone metastases.
